# Alpinumisoflavone Activates Disruption of Calcium Homeostasis, Mitochondria and Autophagosome to Suppress Development of Endometriosis

**DOI:** 10.3390/antiox12071324

**Published:** 2023-06-22

**Authors:** Jisoo Song, Jiyeon Ham, Sunwoo Park, Soo Jin Park, Hee Seung Kim, Gwonhwa Song, Whasun Lim

**Affiliations:** 1 Department of Biological Sciences, College of Science, Sungkyunkwan University, Suwon 16419, Republic of Korea; songjs9251@g.skku.edu; 2 Institute of Animal Molecular Biotechnology, Department of Biotechnology, College of Life Sciences and Biotechnology, Korea University, Seoul 02841, Republic of Korea; glorijy76@korea.ac.kr; 3 Department of Plant & Biomaterials Science, Gyeongsang National University, Jinju-si 52725, Republic of Korea; sw.park@gnu.ac.kr; 4 Department of GreenBio Science, Gyeongsang National University, Jinju-si 52725, Republic of Korea; 5 Department of Obstetrics and Gynecology, Seoul National University College of Medicine, Seoul 03080, Republic of Korea; soojin.mdpark@gmail.com (S.J.P.); bboddi0311@snu.ac.kr (H.S.K.)

**Keywords:** endometriosis, alpinumisoflavone, calcium, autophagy, mitochondria dysfunction

## Abstract

Alpinumisoflavone is an isoflavonoid extracted from the *Cudrania tricuspidate* fruit and *Genista pichisermolliana*. It has various physiological functions, such as anti-inflammation, anti-proliferation, and apoptosis, in malignant tumors. However, the effect of alpinumisoflavone is still not known in chronic diseases and other benign reproductive diseases, such as endometriosis. In this study, we examined the cell death effects of alpinumisoflavone on the endometriosis cell lines, End1/E6E7 and VK2/E6E7. Results indicated that alpinumisoflavone inhibited cell migration and proliferation and led to cell cycle arrest, depolarization of mitochondria membrane potential, apoptosis, and disruption of calcium homeostasis in the endometriosis cell lines. However, the cellular proliferation of normal uterine epithelial cells was not changed by alpinumisoflavone. The alteration in Ca^2+^ levels was estimated in fluo-4 AM-stained End1/E6E7 and VK2/E6E7 cells after alpinumisoflavone treatment with or without calcium inhibitor, 2-aminoethoxydiphenyl borate (2-APB). The results indicated that a combination of alpinumisoflavone and a calcium inhibitor reduced the calcium accumulation in the cytosol of endometriosis cells. Additionally, alpinumisoflavone decreased oxidative phosphorylation (OXPHOS) in the endometriotic cells. Moreover, protein expression analysis revealed that alpinumisoflavone inactivated AKT signaling pathways, whereas it increased MAPK, ER stress, and autophagy regulatory proteins in End1/E6E7 and VK2/E6E7 cell lines. In summary, our results suggested that alpinumisoflavone could be a promising effective management agent or an adjuvant therapy for benign disease endometriosis.

## 1. Introduction

Endometriosis is a chronic debilitating gynecological disorder affecting 5–10% of females of reproductive age [1]. It is a disease characterized by the presence of abnormal endometrium-like tissue outside of the female reproductive tract. Some of the associated clinical symptoms are bowel dysfunction, pelvic pain, infertility, absence of menses, and dysmenorrhea, which result in decreased quality of life of the patient [2,3]. In addition, diagnosis of endometriosis is difficult, and its prevalence is increasing in females of reproductive age [4]. In general, pathological retrograde menstrual theory has been accepted for endometriosis incidence, but the causes of endometriosis are yet to be established clearly. Therefore, therapeutic approaches for treating the disease are focused on reducing the symptoms through surgery, hormone therapy, and inhibition of inflammation; however, the management of endometriosis has several limitations, including the side effects of hormone therapy and surgery. Thus, the development of novel therapeutic agents is required for more efficient management of endometriosis.

Alpinumisoflavone, one of the natural antioxidants, is known as a prenylated isoflavonoid, which is extracted from *Cudrania tricuspidate* fruit and *Genista pichisermolliana*. The physiological effects of alpinumisoflavone, such as in the inhibition of progression of tumorigenesis, have been widely reported in several previous studies [5,6]. Representatively, alpinumisoflavone induces NLRP3 inflammasome-meditated pyroptosis in hepatocellular carcinoma [7]. Also, it suppresses the lesion size of tumor and invasive properties in clear-cell renal cell carcinoma by regulating RLIP76-miR-101 signaling pathways [8]. In a previous study, we identified that alpinumisoflavone disrupted mitochondrial energetics and redox homeostasis, leading to the death of liver cancer cells [5]. Although alpinumisoflavone, which belongs to a class of isoflavonoids with diverse efficacy, has been proven effective in malignant tumors, such as hepatocellular carcinoma, clear-cell renal cell carcinoma and ovarian cancer, via induction of oxidative stress, a study to identify mechanisms related to endometriosis, a benign reproductive disease, has not been reported yet. 

The aim of our study was to ascertain If alpinumisoflavone could regulate cell proliferation and cause apoptosis in endometriosis cell lines. Furthermore, the effect of alpinumisoflavone-induced mitochondria-mediated apoptosis, dysregulation of calcium homeostasis, oxidative phosphorylation, ROS, ER stress, and autophagy in human endometriosis cells was studied.

## 2. Materials and Methods

### 2.1. Compounds

Alpinumisoflavone (Cat No.: CFN98440) was obtained from Chem Faces (Wuhan, China). 2-APB was purchased from Sigma-Aldrich (Cat No.: D9754, St. Louis, MO, USA). The antibodies used in Western blot assay are listed in Table S1.

### 2.2. Cell Culture

End1/E6E7 and VK2/E6E7 cells were obtained from the American Type Culture Collection (ATCC; Manassas, VA, USA). The detailed information about cell maintenance and culture medium have been described in our previous study [2]. The normal uterine epithelial cells were cultured in ReproLife^TM^ reproductive medium (Lifeline Cell Technology; Frederick, MD, USA) in accordance with the instructions and incubated at 37 ℃ in a 5% CO_2_ incubator.

### 2.3. Detection of Cell Proliferative Capacity

The bromo-2′-deoxyuridine (BrdU) ELISA kit (Cat No.: 11647229001, Roche, Basel, Switzerland) was used to assess the proliferative capacity of End1/E6E7 and VK2/E6E7 cells in accordance with the instructions [2,9]. Briefly, End1/E6E7 and VK2/E6E7 cells treated with alpinumisoflavone in a dose-dependent manner (0, 20, and 50 μM) for 48 h were incubated with 10 μM of BrdU. After fixation, anti-BrdU-POD was also incubated for fluorescence visualization. The relative absorbance between the vehicle and treated groups was detected by microplate spectrophotometer (370/492 nm).

### 2.4. Cell Cycle Assay

The cells treated with alpinumisoflavone (0, 20, and 50 μM) for 48 h were washed and fixed with 0.1% BSA-PBS and chilled in 70% ethanol. The fixed cells were collected again and treated with RNase A (Sigma-Aldrich) and propidium iodide (PI; BD Biosciences, Franklin Lakes, NJ, USA) for detection of cell cycle phase (sub G1, G1, S, and G2/M). The results were examined with flow cytometry and this assay was performed independently in triplicate. The detailed process has been described in our previous studies [2,9].

### 2.5. Cell Migration

The End1/E6E7 and VK2/E6E7 cells were seeded in a culture-insert 2 well in μ-dish (Cat No.: 80206, Ibidi GmbH, Munich, Germany). After cell adherence, the inserts were removed and treated with alpinumisoflavone (50 μM). The gap between cells were captured and analyzed using the DM3000 microscope (Leica, Wetzlar, Germany) and Image J software (version 1.53). The detailed process has been described in our previous studies [2,9].

### 2.6. Detection of Apoptotic Cells

Annexin V apoptosis detection Kit I (BD Biosciences) was purchased for the detection of apoptotic cells among End1/E6E7 and VK2/E6E7 cells and used in accordance with the instructions provided by the manufacturer [2,9]. Briefly, the harvested cells treated with alpinumisoflavone (0, 20, and 50 μM) were stained with Annexin V-FITC and PI. The results were measured with BD FACSCalibur Flow Cytometer (BD Biosciences).

### 2.7. Disruption of Mitochondria Membrane Polarity

Depolarization of mitochondrial membrane in endometriotic cells was measured using JC-1 dye as described in our previous study [2,9,10]. The alpinumisoflavone-treated cells were collected and then stained with JC-1 staining solution. After several washing steps, the fluorescence changes from JC-1 polymer to monomer in stained cells were analyzed using BD FACSCalibur Flow Cytometer (BD Biosciences).

### 2.8. Intracellular Calcium Ion Measurement

Intracellular Ca^2+^ levels were measured using Fluo-4 AM dye in accordance with the instructions [2,9,11]. Briefly, alpinumisoflavone-treated endometriosis cells were stained with of Fluo-4 AM (3 μM) for 20 min. The cells were collected again and washed with PBS. After that, the fluorescence changes of cells were detected using BD FACSCalibur Flow Cytometer (BD Biosciences).

### 2.9. Seahorse XFe24 Mito Stress Assay

The End1/E6E7 and VK2/E6E7 cells were seeded in XFe24 cell culture microplates at a density of 60,000 cells/well. After the cells stably adhered to the plate, they were treated with alpinumisoflavone (50 μM) for 7 h at 37 °C in a 5% CO_2_ incubator. Subsequently, oligomycin (1.5 μM), FCCP (0.5 μM), rotenone, and antimycin A (0.5 μM) in the Mito Stress kit were serially administered to evaluate the changes in the oxygen consumption rate (OCR) of the oxidative phosphorylation (OXPHOS) process using a Seahorse XFe 24 analyzer obtained from Agilent Technologies, Inc. All steps were performed according to the manufacturer’s instructions.

### 2.10. Intramitochondrial Calcium Ion Measurement

Intramitochondrial calcium ions were detected using 3 μM Rhod-2AM (Invitrogen, Carlsbad, CA, USA) in accordance with the instructions [2,9]. Briefly, endometriosis cells were treated with alpinumisoflavone for 48 h and subsequently stained with Rhod-2 AM for 30 min. Then, the cells were washed with Hank’s balanced salt solution (HBSS; Gibco) and incubated for 10 min. The results were analyzed with BD FACSCalibur (BD Biosciences).

### 2.11. Immunoblotting

In order to determine the relative protein expression changes by alpinumisoflavone in endometriosis cells, we performed Western blots. Alpinumisoflavone (0, 20, and 50 μM) was treated for 2 h and extracted by whole lysate buffer. For the measurement of protein concentration, the Bradford agent (Bio-Red, Hercules, CA, USA) was used with BSA as the reference. The detailed process regarding loading gel, membrane, and analysis methods has been described in our previous studies [2,9,12].

### 2.12. Statistical Analysis

The general linear model (PROC-GLM) of the SAS software (9.4 version, SAS Institute, Cary, NC, USA) was used to analyze the data in order to determine whether there were any significant differences between vehicle or naïve and treated groups in various cell types. Differences with a probability value of *p* < 0.05 were considered to be statistically significant between various treatment groups and indicated as asterisk marks (* *p* < 0.05, ** *p* < 0.01, *** *p* < 0.001). All assays were independently replicated and data are presented as mean ± standard error of the mean unless otherwise stated.

## 3. Results

### 3.1. Alpinumisoflavone Suppressed Cell Proliferation and Migration in Human Endometriosis-like Cell Lines

**Figure 1:**
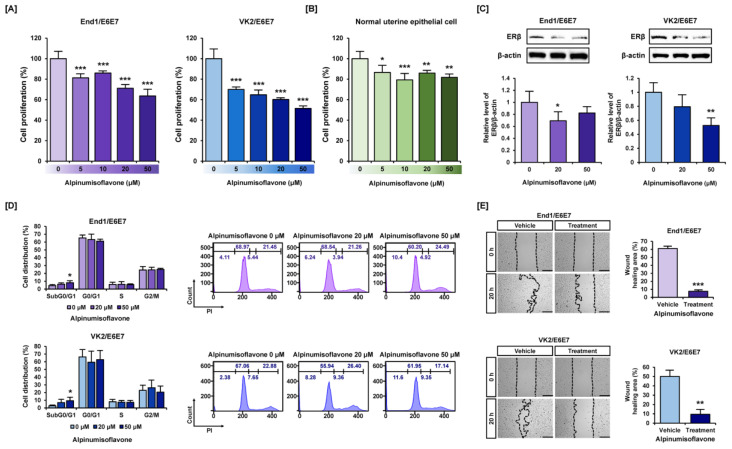
Suppression of cell proliferation and migration by alpinumisoflavone in End1/E6E7 and VK2 E6E7 cells. (**A**) Proliferative capacity of End1/E6E7 and VK2/E6E7 cells with various concentrations of alpinumisoflavone (0, 2, 5, 10, 20, and 50 μM) was analyzed. (**B**) Proliferative capacity of normal uterine epithelial cells treated with alpinumisoflavone. (**C**) Relative protein expressions of ERβ by alpinumisoflavone in End1/E6E7 and VK2/E6E7 cells. (**D**) Changes in cell cycle population were examined in End1/E6E7 and VK2/E6E7 cells with various concentrations of alpinumisoflavone. (**E**) Relative wounded healing area was analyzed using Image J software (version 1.53). The area of vehicle at 0 h was used as a reference. Asterisks indicate significant differences between vehicle and treated groups. All experiments were performed three times, independently (* *p* < 0.05, ** *p* < 0.01, *** *p* < 0.001).

We validated the changes in cell proliferation in human endometriosis-like cell lines End1/E6E7 and VK2/E6E7 treated with various concentrations of alpinumisoflavone (0, 5, 10, 20, and 50 μM) in comparison to normal uterine epithelial cells. Our results showed that the proliferation of End1/E6E7 and VK2/E6E7 cells was reduced to 64% and 51% by 50 μM of alpinumisoflavone, respectively, compared to vehicle (Figure 1A). In contrast, only 18% of cell proliferation was decreased by alpinumisoflavone in primary human normal uterine epithelial cells, which suggests the specificity of alpinumisoflavone for diseased cell types (Figure 1B). We have also verified that alpinumisoflavones cause a reduction in the expression of estrogen receptor beta (ERβ), which is known to be involved in the development of endometriosis, in both End1/E6E7 and VK2/E6E7 cells (Figure 1C). In addition, we used propidium iodide (PI) to analyze alpinumisoflavone-mediated cell cycle arrest in both normal and alpinumisoflavone-treated endometriosis cells. As the concentration of alpinumisoflavone (0, 20, and 50 μM) increased, the cell population in the Sub G0/G1 phase gradually increased, whereas the G0/G1-phase cell population decreased in both End1/E6E7 and VK2/E6E7 cells (Figure 1D). Furthermore, the wounded area recovered to 61% and 50% during 20 h compared to the vehicle group at 0 h in both endometriosis cell lines (Figure 1D). Under alpinumisoflavone (50 μM) treatment, only 8% and 9% were recovered during the same time in End1/E6E7 and VK2/E6E7 cells (Figure 1E, Figure S2). These results revealed that alpinumisoflavone reduced the cell proliferation and migration of human endometriosis-like cells.

### 3.2. Alpinumisoflavone Caused Cell Death and Depolarized Mitochondria Membrane Potential (MMP) in Human Endometriosis Cells

**Figure 2:**
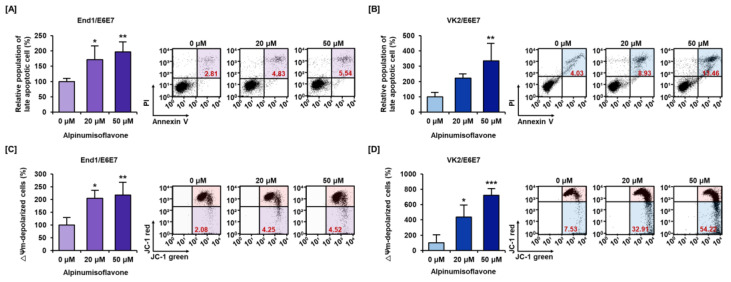
Induction of apoptotic cell death and depolarization of mitochondrial membrane potential by alpinumisoflavone in both endometriosis cell lines. (**A**,**B**) Annexin V and PI staining was used to confirm Annexin V(+)/PI(+) cells, which indicate late apoptotic cells of endometriosis via BD FACSCalibur. The late apoptotic cell population was located in the colored quadrant. (**C**,**D**) Effect of alpinumisoflavone on changes of mitochondria membrane potential (MMP) was measured in End1/E6E7 cells and VK2/E6E7cells. The cell population located in lower right quadrants indicated JC-1 monomer (green fluorescence) and the ratio of relative changes were indicated as bar graphs. All experiments were performed three times, independently (* *p* < 0.05, ** *p* < 0.01, *** *p* < 0.001).

Next, we analyzed the effect of alpinumisoflavone on the induction of apoptotic cell death and depolarization of mitochondria membrane potential (MMP) in End1/E6E7 and VK2/E6E7 cells. It was observed that apoptotic cell death gradually increased to 197% and 334% in End1/E6E7 and VK2/E6E7 cell lines with alpinumisoflavone (0, 20, and 50 μM) treatment, respectively (Figure 2A,B). In addition, mitochondria membrane potential (Δψ) was depolarized to 217% and 720% in End1/E6E6 and VK2/E6E7 cells, respectively, by alpinumisoflavone treatment compared to that in the vehicle group (Figure 2C,D). Thus, these results indicated that cell death triggered by alpinumisoflavone were related to mitochondria in endometriosis cell lines.

### 3.3. Alpinumisoflavone Disrupted Calcium Homeostasis in Cytosolic and Mitochondrial Matrix in End1/E6E7 and VK2/E6E7 Cells

**Figure 3:**
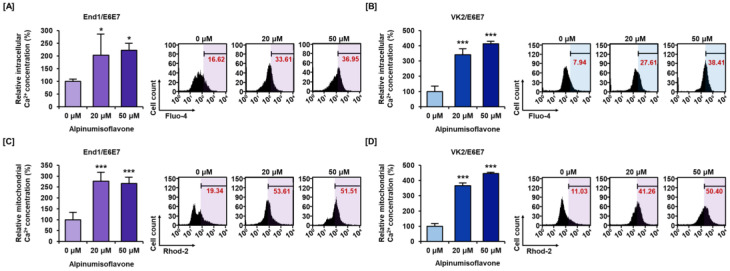
Dysregulation of intracellular calcium ion and mitochondrial calcium ion by alpinumisoflavone in End1/E6E7 and VK2/E6E7 cells. (**A**,**B**) Intracellular calcium ion accumulation was analyzed using Fluo-4 AM. The Fluo-4 (+) population was analyzed. (**C**,**D**) Accumulation of mitochondrial calcium ion was detected by Rhod-2 AM dye. The Rhod-2 (+) population was measured and relative percentage ratio compared to control represented as bar graph. Asterisk marks indicate significant levels.

**Figure 4:**
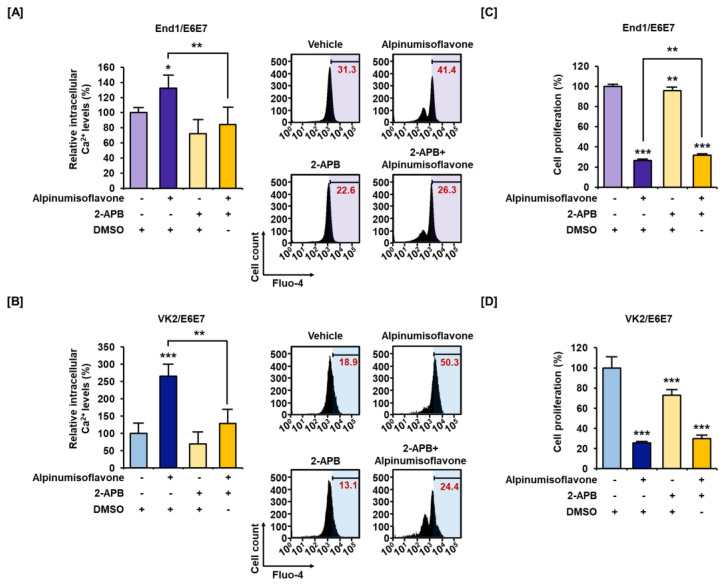
Alleviation effect of alpinumisoflavone on calcium ion dysregulation in human-like endometriosis cells. (**A**,**B**) The endometriosis cell lines were co-treated with alpinumisoflavone (50 μM) for 48 h and with or without 2-APB (2 μM). (**C**,**D**) Relative proliferative capacity of End1/E6E7 and VK2/E6E7 cells treated with or without alpinumisoflavone (50 μM) and 2-APB (2 μM) for 48 h. The absorbance was detected using a microplate spectrophotometer. All experiments were performed three times, independently (* *p* < 0.05, ** *p* < 0.01, *** *p* < 0.001).

Disruption of calcium homeostasis is known to be associated with mitochondrial dysfunction. Therefore, we measured calcium homeostasis of alpinumisoflavone-treated End1/E6E7 and VK2/E6E7 cell lines using Fluo-4 AM and Rhod-2 AM dyes. The intracellular calcium level increased up to 222% and 412% by alpinumisoflavone treatment in End1/E6E7 and VK2/E6E7 cell lines, respectively (Figure 3A,B). Similarly, the mitochondrial matrix calcium level also gradually increased up to 266% and 446% in End1/E6E7 cells and VK2/E6E7 cells, respectively (Figure 3C,D). Through these results, we established that alpinumisoflavone disrupted calcium homeostasis. Additionally, we confirmed calcium accumulation and its effect on cell proliferation through combined treatment with calcium inhibitor 2-APB. Relative cytosolic calcium levels were alleviated from 132% to 84% in End1/E6E7 cells and from 266% to 129% in VK2/E6E7 cells upon co-treatment with 2-APB and alpinumisoflavone (Figure 4A,B). Furthermore, co-treatment slightly restored cell proliferation that was suppressed significantly by alpinumisoflavone treatment alone in human endometriosis cells (Figure 4C,D). Thus, these results indicated that alpinumisoflavone disrupted calcium homeostasis. Additionally, dysregulation of calcium ion levels might be one of the factors that causes cell death; however, it might not be a major reason in the case of endometriosis.

### 3.4. Alpinumisoflavone Regulates Mitochondrial Respiration in End1/E6E7 and VK2/E6E7 Cells

**Figure 5:**
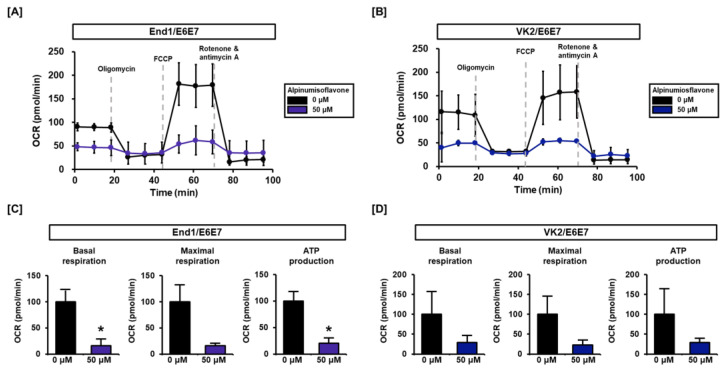
Effect of alpinumisoflavone on mitochondrial respiration in End1/E6E7 and VK2/E6E7 cells. (**A**,**B**) Mitochondrial respiration was measured in human endometriosis-like cells with a Seahorse XFe analyzer. (**C**,**D**) Each stage of basal respiration, maximal respiration, and ATP production influenced by alpinumisoflavone is indicated as a bar graph. Asterisks denote significant differences.

In this study, we investigated the regulation of mitochondrial respiration by alpinumisoflavone in human endometriosis-like cells. Our results reveal that alpinumisoflavone affects mitochondrial respiratory rates at each stage (Figure 5A,B). Prior to oligomycin treatment, the basal respiration of alpinumisoflavone-treated cells was lower than that of vehicle-treated cells. Likewise, the relative OCR of maximal respiration after FCCP treatment was substantially lower with alpinumisoflavone in both cell lines. Additionally, ATP production was reduced by alpinumisoflavone treatment in both cell lines (Figure 5C,D). Therefore, we have demonstrated that alpinumisoflavone disrupts calcium homeostasis and interrupts the electron transfer system of mitochondria, leading to a blockage in the energy supply.

### 3.5. Alpinumisoflavone Downregulates the Intracellular Signaling Pathways Like PI3K/AKT and MAPK in Human Endometriosis-like Cells

**Figure 6:**
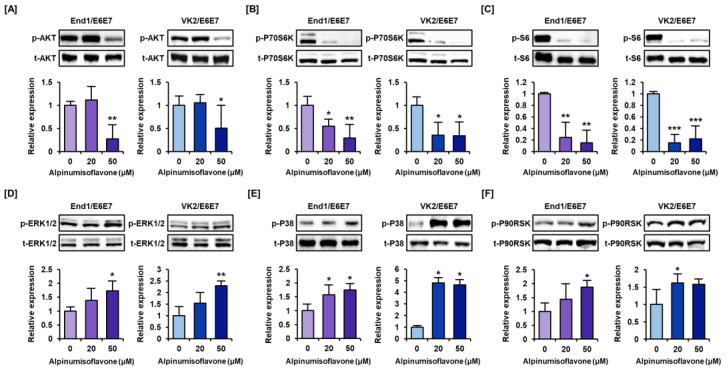
Alpinumisoflavone regulates the PI3K/AKT and MAPK pathway in End1/E6E7 and VK2/E6E7 cells. (**A**–**E**) Immunoblots of phosphor- and total-AKT (**A**), P70S6K (**B**), S6 (**C**), ERK1/2 (**D**), P38 (**E**), and P90RSK (**F**) in response to alpinumisoflavone treatment. Each immunoblot intensity was captured and normalized by each total protein type. Asterisks indicate significant differences between naïve and treatment groups. All experiments were performed three times, independently (* *p* < 0.05, ** *p* < 0.01, *** *p* < 0.001).

The phosphorylation of proteins involved in PI3K/AKT/MAPK pathways, which have been associated with survival of endometriosis, were measured through Western blot assay of alpinumisoflavone-treated endometriosis cell (Figure 6). The phosphorylation of the AKT pathway proteins was decreased to less than half of the control group as the concentration of alpinumisoflavone increased in both End1/E6E7 and VK2/E6E7 cells (Figure 6A). Also, the levels of phosphorylated P70S6K and S6 proteins, downstream of the PI3K/AKT pathway, decreased to less than one-third of that in the control in both cells (Figure 6B,C). However, the phosphorylation of ERK1/2 MAPK increased up to 1.7- and 2.3-fold by alpinumisoflavone treatment in endometriosis cells (Figure 6D). Further, alpinumisoflavone treatment increased phosphor-P38 MAPK and P90RSK proteins significantly (Figure 6E,F). These results confirmed that alpinumisoflavone regulates the phosphorylation of PI3K/AKT and MAPK proteins in End1/E6E7 and VK2/E6E7 cells.

### 3.6. Alpinumisoflavone Regulated the ER Stress and Autophagy Signaling Pathway in End1/E6E7 and VK2/E6E7 Cells

**Figure 7:**
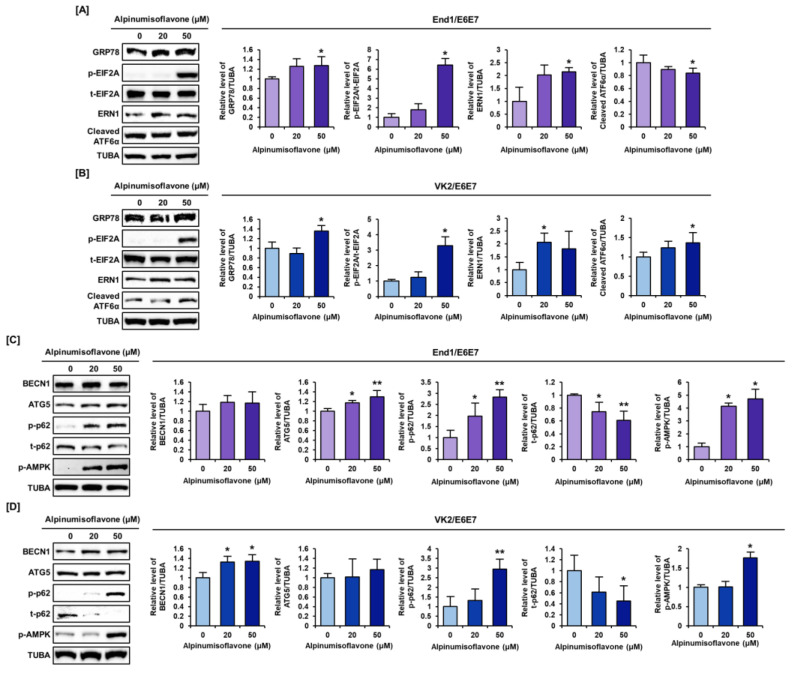
Activation of ER stress and autophagy signal transduction by alpinumisoflavone in human endometriosis cell lines. (**A**,**B**) Immunoblots related to ER stress (GRP78, EIF2A, ERN1, and cleaved ATF6α) and changes in their expression upon treatment with alpinumisoflavone in both endometriosis cells (End1/E6E7 and VK2/E6E7). (**C**,**D**) Immunoblots related to autophagy (BECN1, phosphor-AMPK, ATG5, phosphor-p62, and total-p62) and changes in their expression by alpinumisoflavone treatment in endometriosis cells. TUBA was used for normalization with other proteins. All experiments were performed three times, independently (* *p* < 0.05, ** *p* < 0.01).

We examined the protein level changes related to ER stress and autophagy signaling pathway by alpinumisoflavone (Figure 7). GRP78 protein levels gradually increased by 1.3- and 1.4-fold under alpinumisoflavone treatment for 2 h in End1/E6E7 and VK2/E6E7 cells, respectively (Figure 7A,B). Also, the phosphor-EIF2A and ERN1 protein levels significantly increased by at least two- to six-fold in response to alpinumisoflavone treatment (Figure 7A,B). It was seen that the cleaved form of ATF6α increased slightly in VK2/E6E7 cells, but not in End/1E6E7 cells (Figure 7A,B). Because ER stress is known to trigger autophagy, we also confirmed the changes in protein expression involved in autophagy process. Overall, the protein expression of p-AMPK, Beclin1 (BECN1), and ATG5 tended to increase in both alpinumisoflavone-treated cells, but only ATG5 of End1/E6E7 cells and BECN1 of VK2/E6E7 cells were found to change significantly (Figure 7C,D). Also, the phosphorylation of p62 in Ser^349^ residue drastically increased up to approximately three-fold compared to that in control in both End1/E6E7 and VK2/E6E7 cells (Figure 7C,D). In contrast, the number of p62 proteins gradually reduced to almost half of control in both endometriosis cells, which might suggest the degradation of p62 protein (Figure 7C,D). In conclusion, our results demonstrated the induction of ER stress and autophagy by alpinumisoflavone in both endometriosis cells.

## 4. Discussion

Endometriosis is a chronic disease of females of reproductive age. The cause of this disease is still not clear. However, the prevalence of endometriosis is increasing. A complete diagnosis of endometriosis takes a long time. In addition, the effective management of endometriosis is still insufficient. Therefore, a novel and effective management method of endometriosis is required.

In endometriotic lesions, estradiol primarily expresses ERβ, whereas the normal endometrium predominantly expresses estrogen receptor alpha (ERα) [13]. Phytoestrogens have estrogen-like activity and have been shown to have preventive and therapeutic effects on menopausal syndrome, cardiovascular disease, osteoporosis, and cancer [14]. Because endometriosis is an estrogen-dependent disease, the use of phytoestrogens may exacerbate the disease due to their estrogenic effect. Alpinumisoflavone, a typical phytoestrogen, binds weakly to ERα and ERβ [15,16]. In ovariectomized Wistar rats, alpinumisoflavone administration has estrogenic effects, such as thickening of the uterine epithelium and increased vaginal epithelial height [17,18]. However, in our previous studies, we confirmed the anti-proliferative and therapeutic effects of naringenin and apigenin, which are other types of phytoestrogen, on endometriosis, suggesting that phytoestrogens exhibit mixed agonistic and antagonistic properties [19,20]. Additionally, alpinumisoflavone inhibited the activity of estradiol in human osteosarcoma U2OS cells, which overexpresses both ERα and ERβ. Alpinumisoflavone showed a preference for binding to ERα over ERβ and significantly antagonized the activation of ERβ in the presence of estradiol, indicating an uncommon pattern as a phytoestrogen [21]. Based on several studies, alpinumisoflavone may exhibit anti-estrogenic effects in endometriotic cells and cure them instead of exacerbating the condition.

Recently, among isoflavonoids, alpinumisoflavone has been studied actively by several researchers. However, the studies mainly focused on malignant cancers. Previously, a study established the anti-cancer effect of alpinumisoflavone in ovarian cancer by the disruption of the endoplasmic reticulum (ER) and mitochondria-mediated apoptosis [6]. Another study established that alpinumisoflavone induces apoptosis via modulating miR-370/PIM1 signaling in esophageal squamous cell carcinoma [22]. In the present study, we confirmed the promising therapeutic effects of alpinumisoflavone in both endometriosis cell lines. Like previous studies, our study indicated various changes induced by alpinumisoflavone in the intracellular organelles and intracellular signaling proteins.

It is known that calcium ions form a complex that regulates cell proliferation and death. Calmodulin (CaM) and CaMKII downstream of calcium ions regulate the cell cycle via the cell cycle’s G1, G2/M, and spindle assembly checkpoints [23,24]. As such, calcium ion signals have been identified at various stages of the cell cycle, and elevated cytosolic calcium levels have been seen to activate calcium-sensitive signaling proteins to transmit signals and influence cell survival as well [9,24]. In this study, alpinumisoflavone showed antiproliferative effects along with cell cycle arrest in both endometriotic cell lines. Additionally, Ca^2+^ can induce apoptosis in response to various physiological changes and is generated in both the early and late stages of the apoptosis pathway. Furthermore, calcium ions regulate apoptosis mainly through the level of calcium ions in the ER and mitochondrial systems [25,26]; when the mitochondrial membrane potential is lost, excessive calcium accumulation eventually induces apoptosis [27,28]. Also, the increased mitochondrial calcium ions stimulate oxidative phosphorylation, resulting in increased reactive oxygen species (ROS) production. This, in turn, promotes oxidative stress, mitochondrial permeable transition pore opening, cell death, misfolding of proteins, and proteostatic collapse [29]. Similar to previous studies, alpinumisoflavone was seen to induce mitochondrial membrane potential depolarization and apoptosis in End1/E6E7 and VK2/E6E7. In addition, alpinumisoflavone induced the accumulation of calcium ions in the cytosol and mitochondrial matrix. The effect of alpinumisoflavone on calcium homeostasis was confirmed using calcium inhibitor 2-APB, which is a known antagonist of inositol 1,4,5, -triphosphate (IP3) receptor and suppresses it through membrane penetration of the calcium stores other than mitochondria [30,31]. As a result, calcium accumulation, as well as inhibition of cell proliferation caused by alpinumisoflavone, was alleviated by 2-APB under the same conditions. Therefore, we can conclude that alpinumisoflavone affects cell survival in human endometriotic cells by calcium regulation through the IP3 receptor.

The mitochondrial respiratory chain consists of five complexes located In the inner mitochondrial membrane that synthesize ATP via OXPHOS, along with complex V. Additionally, estrogen can regulate mitochondrial energy-converting capacity and gene expression, modulating ROS levels [32]. Compared to normal endometrial tissues, endometriosis tissue has higher mitochondrial metabolic activity and a higher anti-oxidant defense system, which can better mitigate the intracellular ROS production that results from the high metabolic activity for survival [33]. These activities are the major characteristics of endometriosis pathogenesis, consistent with higher expression of eRβ [34]. Intracellular ROS are mostly generated during the OXPHOS process, with some other sources including the ER and peroxisomes. Although peroxisome function was not directly determined, it is possible that increased ER stress led to increased intracellular ROS, and decreased OXPHOS activity led to a corresponding decrease in mitochondrial ROS. Because the DCFH-DA dye used in our experiments measures intracellular ROS, which was not significantly altered by alpinumisoflavone treatment (Figure S1B,C), we speculated that the ROS increase due to ER stress or peroxisomes was offset by the ROS decrease resulting from decreased OXPHOS. Although we did not observe a substantial increase in ROS that could cause cell death, we believe that the decrease in eRβ (which supplies localized estradiol) and the decrease in OXPHOS (which is a primary energy source for the development of endometriosis) are sufficient to demonstrate the therapeutic effects of alpinumisoflavone. Therefore, alpinumisoflavone suppresses the local supply of estradiol and ATP, thereby interrupting endometriosis development. 

The PI3K and MAPK signaling pathways are well known key regulators of cell survival and apoptosis [35,36]. Some previous studies have shown that regulation of the MAPK pathway and the downregulation of the PI3K-AKT-mTOR axis have confirmed potential therapeutic effects in cancer treatment [37,38,39]; however, more studies on both pathways are required. A previous study showed increased phosphorylated mTOR and AKT in ectopic lesion and benign endometrial disease [40,41]. In addition, our previous research results confirmed the inhibitory effects on cell proliferation by inhibition of the PI3K/AKT pathway [9]. More studies on endometriosis management drugs targeting PI3K/AKT are underway [42,43]. Similar to previous studies, our present study found that alpinumisoflavone downregulates the PI3K/AKT pathway in both endometriosis cell lines. It has also been reported that phosphorylated MAPK via lower expression of TUG1 induced promotion of sensitivity of cisplatin in cervical cancer [44]. Furthermore, *Colocasia esculenta* var. *aquatilis* Hassk extract can induce apoptosis in cervical cancer through enhanced ER stress response and upregulation of c-Jun/P38MAPK protein [45]. Similar to previous studies, in the present study, MAPK pathway proteins were increased in human endometriosis-like cells by alpinumisoflavone.

ER performs key functions, such as stress sensing, signaling function, and regulation of homeostasis in cellular organelles [46,47]. Moreover, ER stress is related to the export of calcium ions to cytosol, and this accumulation of calcium ion in cytosol further leads to regulation of the autophagy and apoptosis pathways [48,49]. The activation of ERN1 (also known as IRE1A) induces ER-mediated mitochondrial apoptosis and suppresses the anti-apoptotic effect of BCL-2 via the activation of MAPK/JNK [50,51]. Phosphorylated EIF2A suppresses protein translation and promotes the expression of GADD43, which is related to growth inhibition [52]. Similar to a previous study, our study confirms that alpinumisoflavone increased the expression of proteins such as GRP78, p-EIF2A, ERN1, and ATF6α.

Autophagy is commonly known as a degradation process for damaged organelles and other cellular components, promoting cell survival or leading to cell death [53,54]. Recently, autophagy has been recognized as a cell death pathway in mammalian systems, where cell death function can be induced via ER stress and downregulation of PI3K/AKT/mTOR [55,56]. In a previous study, resveratrol induced cell death via increased autophagy flux in A549 lung cancer cells, and plasma-activated medium induced autophagic cell death via alteration of the mTOR pathway [57,58]. In addition, phosphorylation of AMPK, which plays an important role in intracellular energy metabolism, can promote the initiation of autophagy by specifically phosphorylating autophagy-related proteins. Activation of AMPK induces autophagy through negative regulation of the mTOR protein kinase complex and activation of ULK1, the initiation marker of autophagy [59,60]. In a previous study, curcumin induced autophagy in the A549 lung adenocarcinoma cell line through phosphorylation of APMK and phosphorylation of acetyl-COA carboxylase [61]. In endometriosis, AMPK is usually downregulated compared to normal cells, inducing aberrant autophagy; therefore, stimulating AMPK would be helpful to activate autophagy in the therapeutic aspect, similar to other drugs such as dienogest and metformin [62,63]. Similarly, this study revealed that alpinumisoflavone increased protein expression of autophagy and increased AMPK phosphorylation. Although ATG5 did not show a significant change in VK2/E6E7 due to alpinumisoflavone, autophagy can be induced independently or non-independently in mammalian cells [64,65].

## 5. Conclusions

In conclusion, our results suggest that alpinumisoflavone has promising potential as a management agent or adjuvant therapy for benign endometriosis. We have elucidated the mechanisms of action of alpinumisoflavones by investigating the function of diverse cellular organelles and redox mechanisms in endometriotic cells. Alpinumisoflavone induces mitochondrial dysfunction and excessive calcium ion accumulation in endometriotic cells. Furthermore, we confirmed a decrease in ERβ and OXPHOS activity, which may induce a reduction of mitochondrial ROS. Although our study has identified the pharmacodynamics and pharmacokinetics of alpinumisoflavone, we have not identified the exact sources of oxidative stress in each organelle at the cellular level and the antioxidant effects of alpinumisoflavone in vivo. Therefore, in the future, it will be necessary to verify the action of alpinumisoflavones in a humanized animal model of endometriosis in vivo. 

## Data Availability

Data are contained within the article.

## Supplementary Materials

The following supporting information can be downloaded at: https://www.mdpi.com/article/10.3390/antiox12071324/s1.
