# Supplementary material for: Alpinumisoflavone Activates Disruption of Calcium Homeostasis, Mitochondria and Autophagosome to Suppress Development of Endometriosis
Source: Antioxidants (Basel). 2023 Jun 22;12(7):1324. doi: 10.3390/antiox12071324 (PMC10376749; doi:10.3390/antiox12071324)
Supplement: Supplementary file 1 [file antioxidants-12-01324-s001.zip › antioxidants-2422540-Supplemental Table S1.pdf]

Supplemental Table S1. List of antibodies used in this study.

Antibody	Cat No.	Sources
p-AKT (Ser^{473})	4060	Cell signaling Technology
p-P70S6K (Thr^{421}/Ser^{424})	9204	Cell signaling Technology
p-S6 (Ser^{235}/Ser^{236})	2211	Cell signaling Technology
p-ERK1/2 (Thr^{202}/Tyr^{204})	9101	Cell signaling Technology
p-P38MAPK (Thr^{180}/Tyr^{182})	4511	Cell signaling Technology
p-P90RSK ($Ser^{\mathbf{573}}$)	9346	Cell signaling Technology
AKT	9272	Cell signaling Technology
P70S6K	9202	Cell signaling Technology
S6	2217	Cell signaling Technology
ERK1/2	4695	Cell signaling Technology
P38MPAK	9212	Cell signaling Technology
RSK1/RSK2/RSK3	9355	Cell signaling Technology
GRP78	sc-13968	Santa Cruz Biotechnology
p-eIF2α (Ser51)	3398	Cell signaling Technology
eIF2α	5324	Cell signaling Technology
IRE1 α	3294	Cell signaling Technology
ATF6 α	sc-166659	Santa Cruz Biotechnology
p-P62 ($Ser^{\mathbf{349}}$)	16177	Cell signaling Technology
P62	88588	Cell signaling Technology
Beclin1	3495	Cell signaling Technology
ATG5	12994	Cell signaling Technology
TUBA	sc-32293	Santa Cruz Biotechnology
