# Supplementary figures and images for: Alpinumisoflavone Activates Disruption of Calcium Homeostasis, Mitochondria and Autophagosome to Suppress Development of Endometriosis
Source: Antioxidants (Basel). 2023 Jun 22;12(7):1324. doi: 10.3390/antiox12071324 (PMC10376749; doi:10.3390/antiox12071324)

[A]

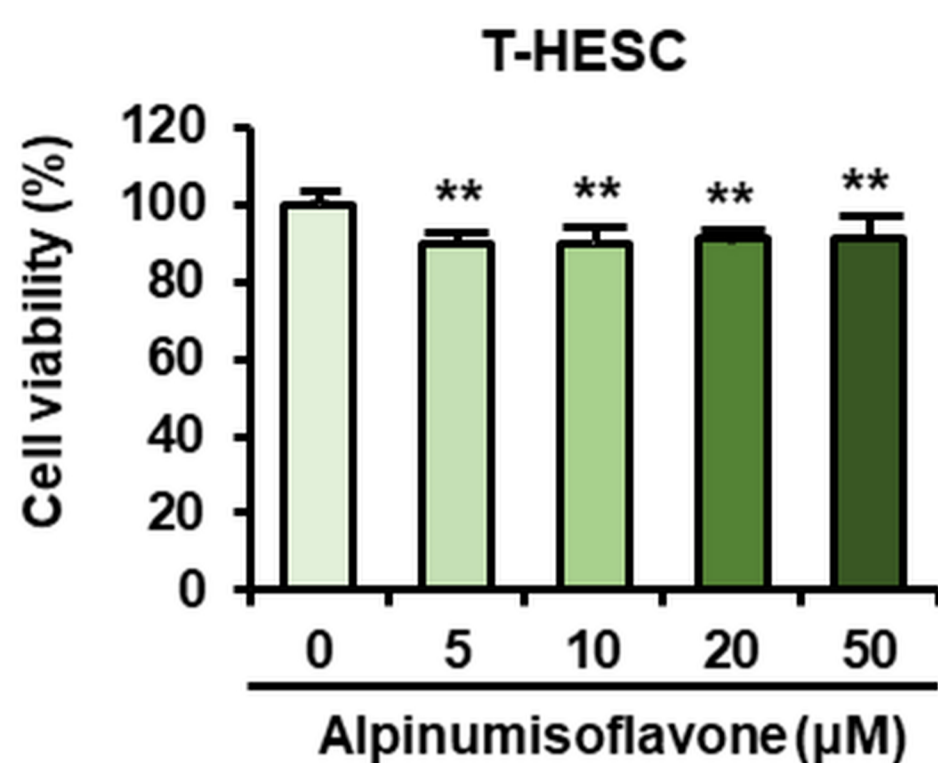

[B]

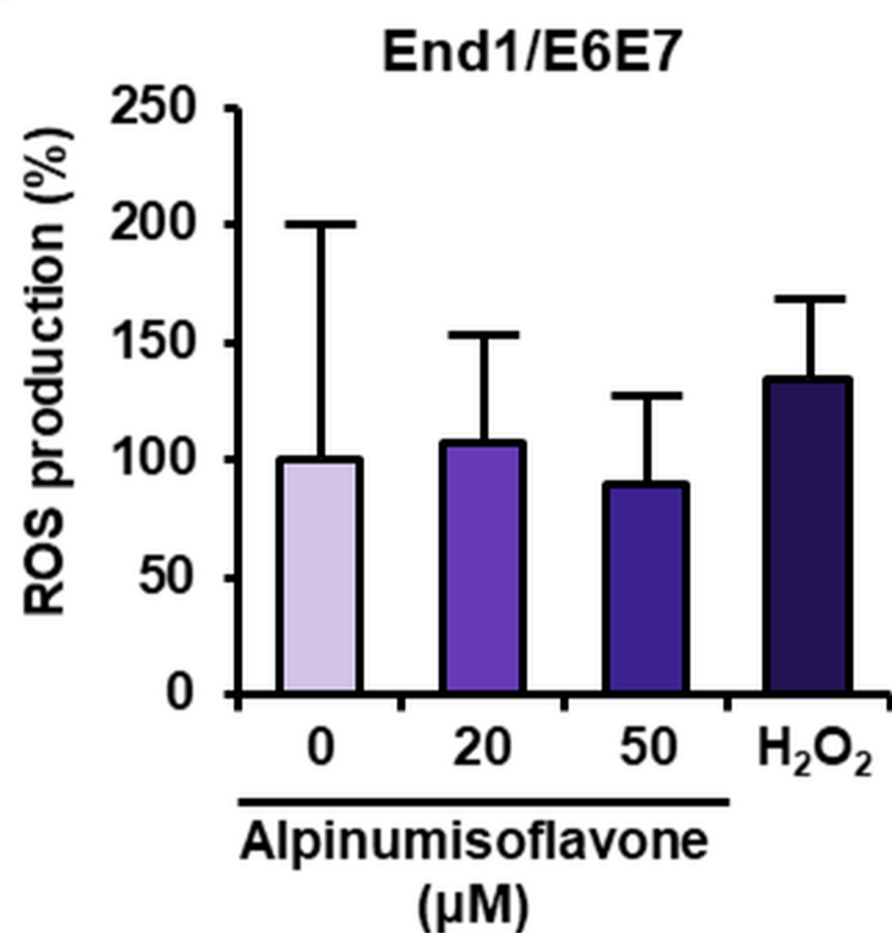

[C]

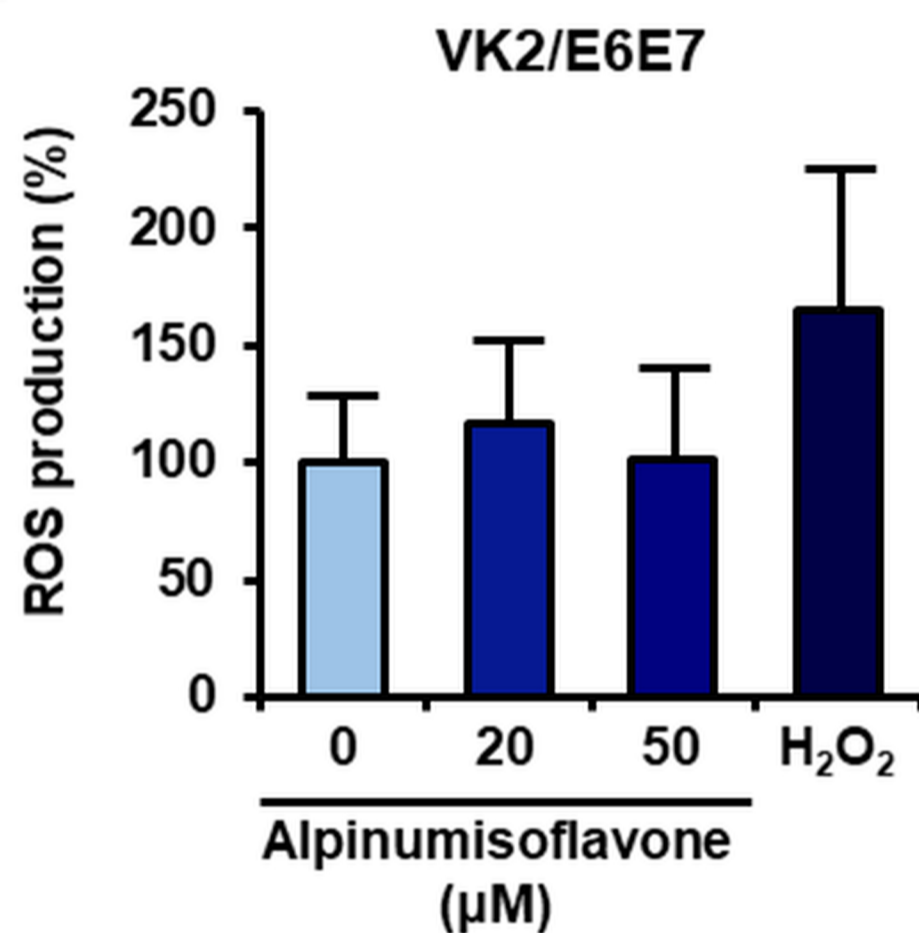

[D]

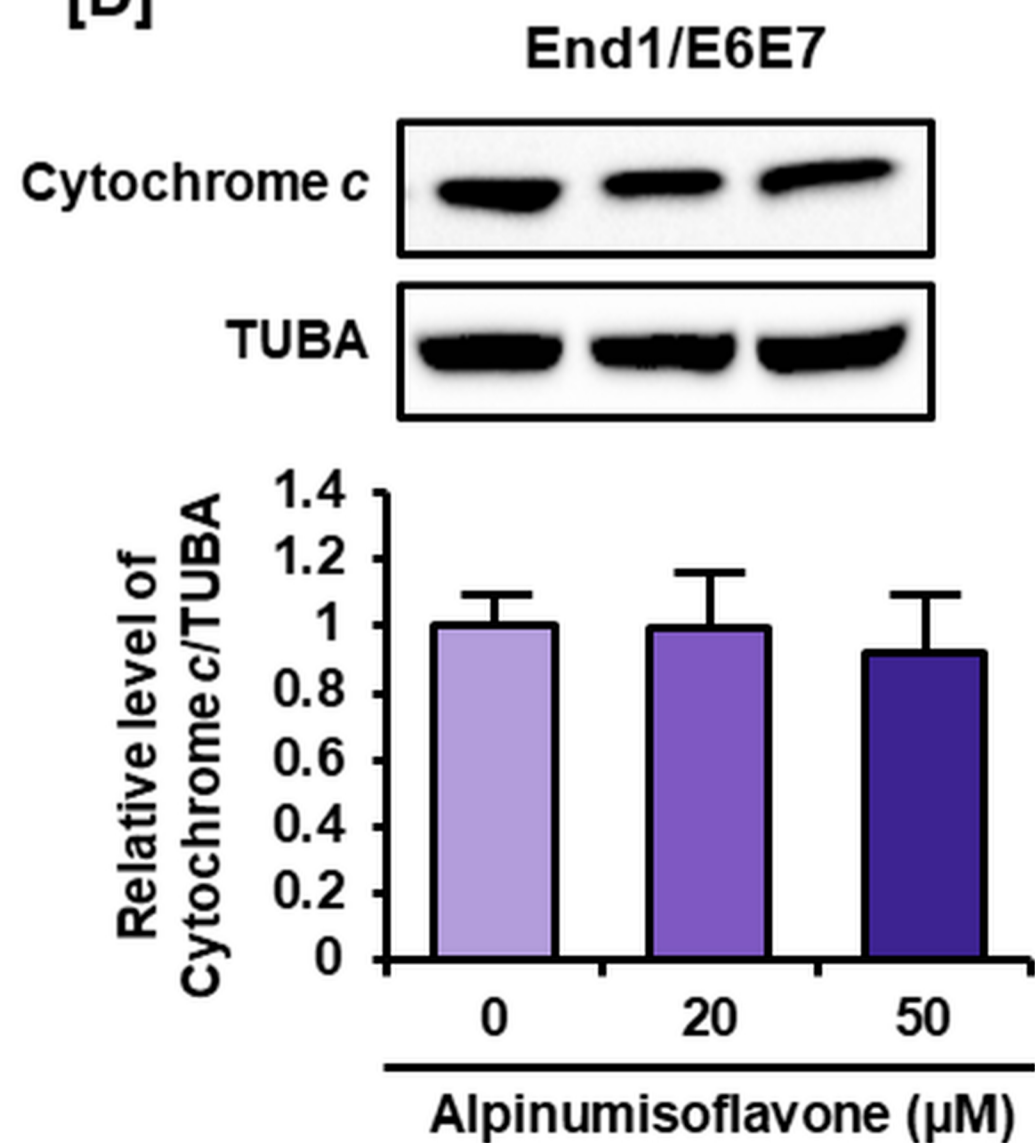

[E]

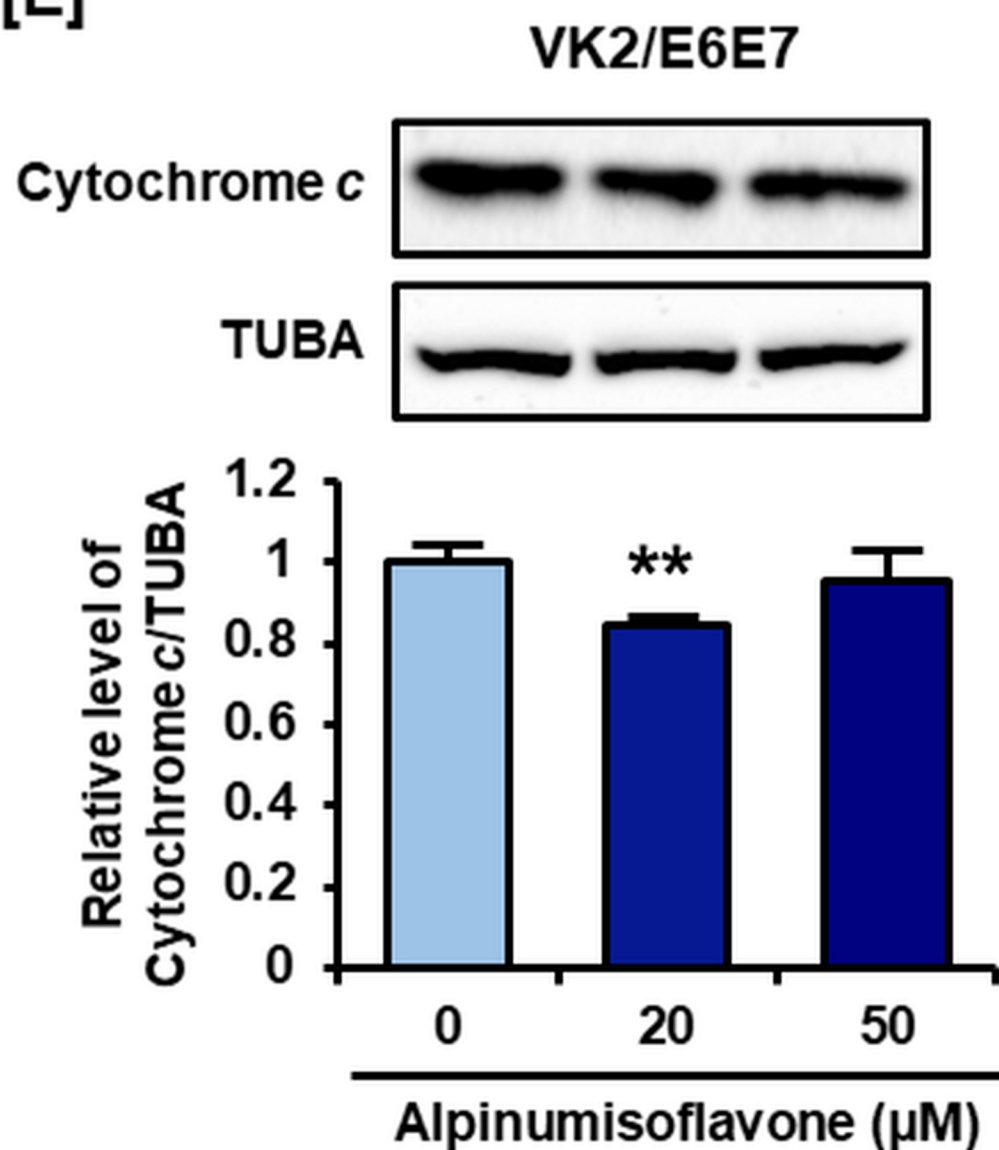

Supplement: Supplementary file 1 [file antioxidants-12-01324-s001.zip › Supplementary Figure S1.pdf]

[A]

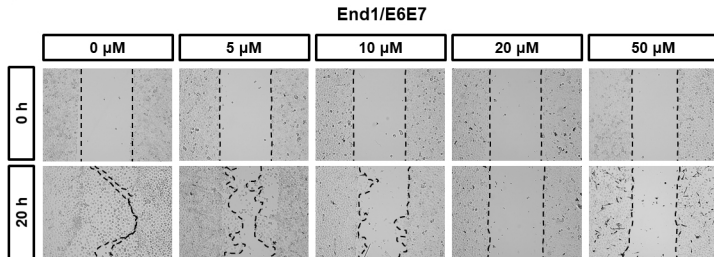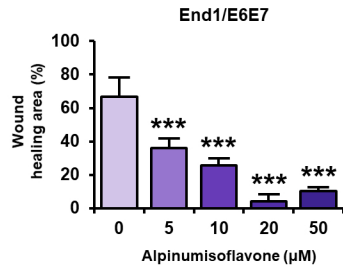

[B]

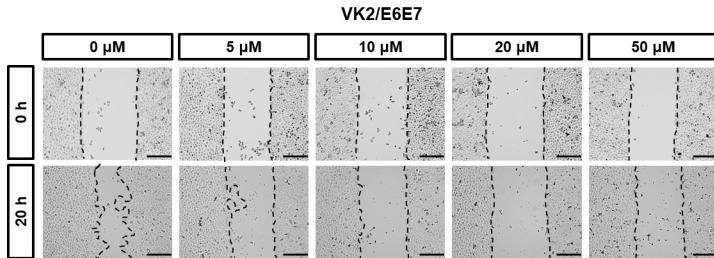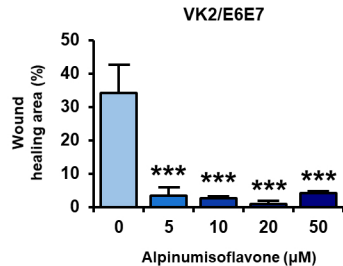

Supplement: Supplementary file 1 [file antioxidants-12-01324-s001.zip › Supplementary Figure S2.pdf]
